# Rab GTPases: Central Coordinators of Membrane Trafficking in Cancer

**DOI:** 10.3389/fcell.2021.648384

**Published:** 2021-06-01

**Authors:** Hongyuan Jin, Yuanxin Tang, Liang Yang, Xueqiang Peng, Bowen Li, Qin Fan, Shibo Wei, Shuo Yang, Xinyu Li, Bo Wu, Mingyao Huang, Shilei Tang, Jingang Liu, Hangyu Li

**Affiliations:** Department of General Surgery, The Fourth Affiliated Hospital of China Medical University, Shenyang, China

**Keywords:** Rab GTPase, membrane trafficking, cancer biology, GEF, GDP

## Abstract

Tumor progression involves invasion, migration, metabolism, autophagy, exosome secretion, and drug resistance. Cargos transported by membrane vesicle trafficking underlie all of these processes. Rab GTPases, which, through coordinated and dynamic intracellular membrane trafficking alongside cytoskeletal pathways, determine the maintenance of homeostasis and a series of cellular functions. The mechanism of vesicle movement regulated by Rab GTPases plays essential roles in cancers. Therefore, targeting Rab GTPases to adjust membrane trafficking has the potential to become a novel way to adjust cancer treatment. In this review, we describe the characteristics of Rab GTPases; in particular, we discuss the role of their activation in the regulation of membrane transport and provide examples of Rab GTPases regulating membrane transport in tumor progression. Finally, we discuss the clinical implications and the potential as a cancer therapeutic target of Rab GTPases.

## Key Points

1. There are 66 Rab GTPases associated with vesicular transport in the human genome.
2. The GEFs and GDPs make Rab GTPases act as molecular switches.
3. Based on membrane trafficking function, Rab GTPases are important to cancer progression.
4. Targeting Rab GTPases provides possibilities for cancer treatment strategies.

## Introduction

Although many studies have strongly supported the notion that the dysregulation of invasion, migration, metabolism, autophagy, exosome secretion and drug resistance mediate cancer progression, few studies have focused on intracellular membrane trafficking, which regulates these processes (Prasad et al., 2016). Membrane trafficking enables the distribution of cellular proteins and the secretion of extracellular vesicles (Kajiho et al., 2018). Notably, the transport of membrane along the cytoskeleton regulates various biological functions in the cell. Membrane trafficking in cancer is crucial to these processes, in which membrane dynamics mediate their physical requirements (Stenmark, 2009). Indeed, variations in the presentation and degradation of important membrane proteins and imbalance in dynamic vesicle trafficking processes are known to be critical in tumor progression (Cho et al., 2018; Drizyte-Miller et al., 2020; Yousaf and Ali, 2020). Thus, membrane trafficking is a focal point for targeting cancer. Membrane trafficking can “drive” cancer progression and enable invasion, migration, metabolism, autophagy, exosome secretion and drug resistance.

Rab GTPases are highly conserved regulators of vesicular transport, and 66 members of this family in the human genome have been described (Li and Marlin, 2015). They switch between an active and inactive state, which is modulated by guanine nucleotide exchange factor (GEF) and GTPase activating protein (GAP), and function via downstream molecules, such as coat proteins and motor proteins, to trigger downstream membrane trafficking (Lamber et al., 2019). Each Rab GTPase is localized to a different membrane compartment, where it controls the specificity and direction of membrane transport (Stenmark, 2009). Rab GTPases regulate different trafficking routes and perform specific tasks in a series of membrane trafficking steps (Minamino and Ueda, 2019). Indeed, Rab GTPases ensure that cargos are transported to the correct target. Through transient interactions with downstream molecules, they control the formation of membrane buds, vesicular transport along the cytoskeleton, and membrane fusion to the target compartment (Cernochova et al., 2016; Burk and Pasterkamp, 2019).

**FIGURE 1:**
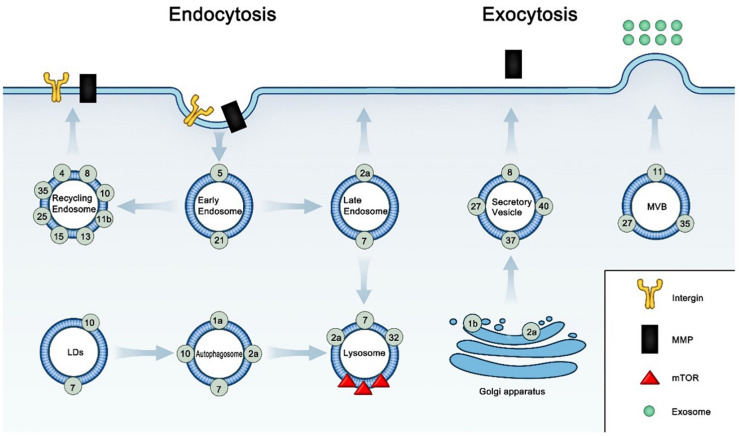
Subcellular localization and relative function of Rab GTPases in cancer.

As cancer cells live in changing microenvironments, they need a class of checkpoints that regulate the balance among endocytosis, recycling, degradation and exocytosis to face external stresses (Yang T. et al., 2020). Rab GTPases play a specific and predominant role in vesicle trafficking (Homma et al., 2020). Research has traditionally been mainly focused on regulation of the transcription or translation of Rab GTPases. Changes in Rab GTPase expression are associated with invasion, migration, metabolism, autophagy, exosome secretion and drug resistance in cancer (Table 1). A number of these changes are caused by changes in vesicular transport pathways, which influences cargo delivery to the cellular membrane and cargo endocytosis, recycling, degradation in lysosomes and exocytosis (Lamber et al., 2019). The checkpoint between recycling and degradation can significantly affect the cell biological function (Cullis et al., 2002; Ceresa and Bahr, 2006). Increases in endocytosis and endocytosed proteins transported to the lysosomes can decrease cell surface proteins (Drizyte-Miller et al., 2020). Indeed, error in the delivery of proteins to the cell surface or lysosomes causes abnormalities in polarity and membrane protein function, which can significantly affect cell invasion, migration and drug resistance. In addition, the overexpression or knockdown of Rab GTPases to reduce exosome transmission affected cancer progression (Hendrix and De Wever, 2013; Li et al., 2013; Huang and Feng, 2017; Chen et al., 2018; Guo et al., 2019). In addition, changes in internal trafficking decisions, including decisions to recycle and degrade through regulating ubiquitinylation and autophagy, can regulate receptor signaling and related protein function (Dong et al., 2019; Drizyte-Miller et al., 2020). In conclusion, the regulation of vesicle trafficking by Rab GTPases can affect receptor recycling and trafficking, which can affect tumor invasion, migration, metabolism, autophagy, exosome secretion and drug resistance. These roles and the subcellular localization of b GTPases are briefly summarized in Figure 1. This review seeks to highlight the potential contributions of Rab GTPase-mediated membrane trafficking to tumor progression, the clinical implications of Rab GTPases and the therapeutic potential of targeting Rab GTPases.

**TABLE 1:** The role of vesicle trafficking medicated by Rab GTPases in cancer research.

**Functions**	**Rab GTPases**	**Regulatory factors**	**Cancer research**	**References**
Invasion	Rab1b	MMP1	Breast/melanoma/colon cancer	Pellinen et al., 2006
	Rab2a	MT1-MMP	Breast cancer	Yuan and Wei, 2021
	Rab4a	Procathepsin L	Melanoma	Halberg et al., 2016
	Rab4/Rab5	MT1-MMP	Breast cancer	Howe et al., 2020
	Rab7	MT1-MMP	Colon cancer	Kajiho et al., 2016
	Rab8	MT1-MMP	Breast cancer	Jeong et al., 2019
	Rab11	Integrin α6β4	Breast cancer	Nam et al., 2010
	Rab25	Integrin α5β1 and β1	Breast cancer	Jeong et al., 2018
	Rab27a	MMP9	Breast cancer	Bravo-Cordero et al., 2007
		Cytokines	Colon cancer	Li et al., 2013
	Rab27b	MMP2	Breast cancer	Yoon et al., 2005
	Rab37	TIMP1	Non-small-cell lung cancer	Argenzio et al., 2014; Sahgal et al., 2019
		TIMP2	Nasopharyngeal carcinoma	Argenzio et al., 2014; Sahgal et al., 2019
	Rab40	MMP2/9	Breast cancer	Wang et al., 2019
Migration	Rab5	Integrin β1	Breast/prostate cancer	Caswell et al., 2007
	Rab5a	Integrin β1	Pancreatic cancer	Jacob et al., 2013
	Rab10	Integrin β1	Cervical cancer	Bobrie et al., 2012
	Rab11b	Integrin β1	Breast cancer	Li et al., 2018
	Rab13	Integrin β1	Cervical cancer	Bobrie et al., 2012
	Rab21	Integrin β1	Breast/prostate cancer	Caswell et al., 2007
	Rab25	Integrin α1/β4/β6	Skin squamous carcinoma	Wang et al., 2018
		Integrin β1	Non-small-cell lung cancer	Steffan et al., 2014; Alonso-Curbelo et al., 2014
		Integrin β1	Ovarian cancer	Steffan et al., 2014; Alonso-Curbelo et al., 2014
		Integrin β1	Colon cancer	Barbarin and Frade, 2011
	Rab35	Integrin β1	Breast/cervical cancer	Hendrix et al., 2010
Growth Factor Driven Signaling of Migration and Invasion	Rab5	EGFR	Breast cancer	Stallaert et al., 2018
	Rab7	HGF	Colon cancer	Kajiho et al., 2016
	Rab10	HGF	Hepatocellular carcinoma	Wang W. et al., 2017
	Rab11	HGF	Gastric cancer	Dong et al., 2016
	Rab11a	EGFR	Breast cancer	Yamori et al., 1981
	Rab31	HGF	Colon Cancer	Yang Y. et al., 2020
Metabolism	Rab32	mTOR	Hepatocellular carcinoma/cervical cancer	Drizyte-Miller et al., 2020
	Rab25	GLUT1	Breast/ovarian cancer	Cheng et al., 2012
Autophagy	Rab1a	Autophagosome	Neuroblastoma	Song et al., 2018
	Rab2/Rab7	Autolysosome	Breast cancer	Lorincz et al., 2017
	Rab2a	Autophagosome	Breast cancer	Ding et al., 2019
	Rab10	LD	Hepatocellular carcinoma	Li et al., 2016; Li et al., 2020b
	Rab7	LD	Hepatocellular carcinoma	Cho et al., 2018
	Rab25	Autophagosome	Ovarian cancer	Liu et al., 2012
	Rab2/Rab7	Autophagosome	Renal carcinoma	Tringali et al., 2012
Exosome	Rab11	Exosome secretion	Leukemia	Savina et al., 2002
		Exosome secretion	Leukemia	Savina et al., 2005
		Exosomal TMPRSS2	Breast cancer	Chi et al., 2020
	Rab27	Exosomal miR-23b	Bladder carcinoma	Ostenfeld et al., 2014
	Rab27a	Exosome secretion (size of MVB)	Ovarian cancer	Ostrowski et al., 2010
		Exosome	Melanoma	Hendrix and De Wever, 2013; Chen et al., 2018; Guo et al., 2019
		Exosome	Breast cancer	Hendrix and De Wever, 2013; Chen et al., 2018; Guo et al., 2019
		Exosome	Colon cancer	Hendrix and De Wever, 2013; Chen et al., 2018; Guo et al., 2019
		Exosome	Colon cancer	Huang and Feng, 2017
		Exosome (antitumor immunity)	Lung cancer	Li et al., 2013
		Exosome (neutrophil mobilization)	Breast cancer	Bravo-Cordero et al., 2007
	Rab27b	Exosome secretion (localization of MVB)	Ovarian cancer	Ostrowski et al., 2010
		Exosomal miR-34c-5p	Leukemia	Peng et al., 2018
	Rab27a/b	Exosome (from macrophage)	Head and neck squamous cell carcinoma	Madeo et al., 2018
	Rab27a/b	Exosomal miR-365	Pancreatic cancer	Binenbaum et al., 2018
Drug resistance	Rab7a	Extracellular vesicle	Cervical cancer	Guerra et al., 2019
	Rab8	TMEM 205	Epidermoid carcinoma	Shen and Gottesman, 2012
		TNFRSF10B	Non-small-cell lung cancer	Wang et al., 2020
	Rab13	EGFR	Gastric cancer	Chen et al., 2019
	Rab5a/Rab21	ABCG2	Breast cancer	Yousaf and Ali, 2020
	Rab27a	Exosome	Ovarian cancer	Dorayappan et al., 2018
				

Active Rab GTPases locate in specific membrane compartments and function in cancer by membrane trafficking, according to Table 1. In the early endosome, Rab5 and Rab21 regulate the endocytosis of integrins and ABCG2 in cell surface. In the recycling endosome, Rab4, Rab8, Rab10, Rab11b, Rab13, Rab25, and Rab35 recycle endosomal integrins, MMPs, EGFR, and TNFRSF10B to the cell surface. In the late endosome, Rab2a control the endocytic recycling of MMT1-MMP; Rab7a affects resistance by increasing the secretion of extracellular vesicle. In the LDs, Rab7 and Rab10 induces the selective autophagy of LDs (lipophagy). In the autophagosome, Rab7 and Rab10 regulate lipophagy; Rab1a and Rab10 control the formation of autophagosome; Rab2 and Rab7 regulate the maturation of autophagosome. In the lysosome, Rab2a functions in autophagosome clearance; Rab7 regulate lipophagy; Rab32 regulate the transport of mTOR signaling protein into lysosome. In Golgi apparatus, Rab1b regulate the secretion of MMP1 from Golgi apparatus; Rab2a control the transport of E-cadherin to the Golgi apparatus. In the secretory vesicle, Rab27, Rab37 and Rab40 control the exocytosis of MMP1, TIMP1, or TIMP2; Rab8 increases the secretion of TMP. In the MVB, Rab11, Rab27, and Rab35 control exosome biogenesis and secretion process.

## Rab GTPase Function in Membrane Trafficking

**FIGURE 2:**
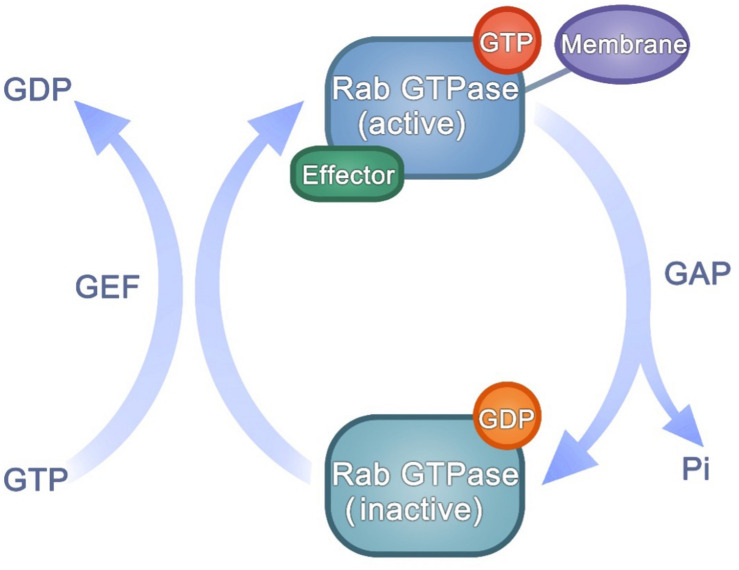
The activation of Rab GTPases.

Many studies have indicated that different Rab GTPases function closely in arranging membrane transport (Stenmark, 2009). These classes of Rab GTPases can define distinct types of membranes and regulate membrane tethering, movement and fate. Rab GTPases are present in an active or inactive state. Their nucleotide-dependent conformational transformation makes Rab GTPases act as molecular switches. The guanine nucleotide-bound state of Rab GTPases is regulated by GAPs or GEFs (Lamber et al., 2019). Rab GTPase contains two switch regions affected by GTP/GDP binding state (Goud et al., 2018). Through specific binding to the two switch regions, GAPs and GEFs control the GTP/GDP cycle (Figure 2). Every GTP/GDP cycle represents an opportunity to adjust the transport direction: to recruit a tether protein or a motor protein and promote traffic, or to adjust downstream proteins from actin filaments to microtubules, or to hand off the regulation of trafficking between Rab-dependent regulators (Malia et al., 2018; Baba et al., 2019; Dolce et al., 2020).

Hence, GAP/GEF determine the quantities of active Rab GTPases. Impairment of GAPs or overexpression of GEFs can cause an increase in active Rab GTPases, influencing the recruitment of downstream effectors regulated by active Rab GTPases, then may regulate cancer progression (Novick, 2016). For example, the eco tropic viral integration site 5 (EVI5), which belongs to a small subfamily of Tre-2/Bub2/Cdc16 (TBC) domain-containing proteins, is an oncogene that regulates the proliferation and metastasis in lung cancer (Cai et al., 2020). And there has been a promising prognosis predicting model for melanoma based on TBC family protein (Tang D. et al., 2020). Furthermore, Rab8 and Rabin8 (the GEF of Rab8) promote tumor formation (Choi et al., 2020).

In addition to GAPs and GEFs, Rab GDP dissociation inhibitors (GDIs) and Rab escort proteins (REPs) also functions in the adjustment of the Rab GTPases activity (Stenmark, 2009; Müller and Goody, 2018). GDIs were initially identified as factors preventing the release of GDP from the Rab GTPases and stabilizing the inactive state of Rab GTPases. And GDIs and GEFs also function as chaperones of Rab GTPases in the cytoplasm. RabIF, one of the GEFs, could stabilize the protein expression of Rab10 (Gulbranson et al., 2017). And two GDIs (GDI1 and GDI2) could regulate the geranylgeranylation of Rab GTPases and thus regulate their delivery and recycle. REPs also modulate the geranylgeranylation of Rab GTPases and affect their targeting in the membrane trafficking.

Some studies indicate that one Rab GTPase can interact with the GEFs or GAPs specific for other Rab GTPases, a critical discovery for their ordered activation in the membrane transport process (Tang L. et al., 2020). After activation, Rab GTPases can interact with multiple effectors in time and space to selectively regulate cargos into vesicles, exert vesicle movement along actin and microtubule cables, and tether vesicles for membrane fusion. For example, Rab GTPase adaptor proteins and lipid kinases and phosphatases assist Rab GTPases in cargo selection and membrane remodeling (Malia et al., 2018; Baba et al., 2019). In addition, Rab GTPases bind motor proteins to regulate vesicular transport along the cytoskeleton and function with tethering proteins in membrane fused with the target compartment (Dolce et al., 2020).

Rab GTPases switch between active and inactive conformations in a GTP-dependent mode. GEF catalyzes GDP-bound to GTP-bound, while GAP promotes the opposite process. The GTP/GDP cycle affects the state of Rab GTPases, and the activated Rab GTPases can bind to downstream effectors and function in regulating membrane trafficking.

## Rab GTPases and Cancer Progression

### Migration and Invasion

Tumor metastasis is inseparable from abnormal cell invasion, proliferation, migration and angiogenesis, among which cell migration and invasion are the most important markers (Gkretsi and Stylianopoulos, 2018). Cell migration and invasion require a class of cell processes, including the intracellular vesicular transport of cell adhesion receptor molecules (such as integrins, cadherin-catenin) and proteases (Castro-Castro et al., 2016; LaFlamme et al., 2018). It has also been determined that ligand-growth factor receptor interactions through membrane transport pathways are associated with two important stages of cancer metastasis: cell invasion and migration. There is no doubt that vesicle trafficking medicated by Rab GTPases plays an important role in these processes.

Knowledge of the Rab GTPases functions in vesicular transport is crucial to understand the mechanisms of migration and invasion. Specifically, cell migration and invasion require the redistribution of adhesive components and the directional delivery of matrix-degrading enzymes.

#### Migration

In the former, integrins trafficked to the cell surface regulate the formation of filopodia and actin-matrix interactions, which can significantly affect cell adhesion and migration. Rab5 and Rab21 regulate the endosomal trafficking of integrin β1 and their overexpression increases the ability of cell migration and adhesion (Pellinen et al., 2006). Rab5a also promotes the formation of filopodia that promote migration in pancreatic cancer cells by activating integrin β1 (Yuan and Wei, 2021). The Rab11 protein family (Rab11b and Rab25) have played a key role in cancer cell migration by regulating integrin recirculation at the migration front. Regulation of integrin β1 surface expression by Rab11b plays a crucial role in cell adhesion, activating Erk signaling and promoting the adaptation and growth of breast cancer brain metastases (Howe et al., 2020). Rab25 can act as a tumor suppressor or tumor promoter in different tumors. Rab25 plays a tumor suppressive role in colorectal cancer and cutaneous squamous cell carcinoma. Rab25 deficiency induced the impairment of integrin β1, β4, and α6 recycling, causing the improper expression of integrins in skin squamous cell carcinoma (Jeong et al., 2019). Besides, loss of Rab25 prominent reductions in integrin β1 and promotes integrin β1 away from the lateral membranes in colon cancer (Nam et al., 2010). By contrast, Rab25 promotes integrin β1 trafficking to the cytoplasmic membrane in non-small-cell lung cancer and ovarian cancer and acts as a tumor promoter (Jeong et al., 2018; Wang et al., 2019). In ovarian cancer, Rab25-medicated integrin β1 activates EGFR/VEGF/Snail axis and then promote cell invasion. And in non-small-cell lung cancer, Rab25-medicated integrin β1 activates AKT/β-catenin pathway. Integrin recycling is also mediated by Rab10 and Rab13. Rab10 and Rab13 interact with Golgi-localized gamma ear-containing Arf-binding protein 2 and facilitate the recycling of active integrin β1 to the cell surface in cervical cancer (Sahgal et al., 2019). Another Rab GTPase controlling the recycling of integrin is Rab35. Rab35 can increase the expression of integrin β1 in cervical and breast cancer and promote cell migration (Argenzio et al., 2014).

#### Invasion

In the latter, tumor cells secrete a variety of proteases, such as MMP, that help cells break through the basement membrane and ECM. Some Rab GTPases have been described in the context of MMP secretion or activation vesicular transport regulates the delivery of MMPs and other proteins to change the extracellular matrix and thereby affect cell invasion. MT1-MMP (Membrane-type 1-matrix metalloproteinase) and other MMPs are essential for matrix remodeling and invasion. For example, Rab1b can regulate metastasis by increasing the secretion of MMP1 (Halberg et al., 2016). Rab2a regulates MT1-MMP endocytic recycling and the transport of E-cadherin to the Golgi to promote cell invasion in breast cancer (Kajiho et al., 2016). Rab5/Rab4 promotes invasion by MT1-MMP in invasive breast cancer (Frittoli et al., 2014). Rab8 controls the exocytosi**s** of MT1-MMP in breast cancer (Bravo-Cordero et al., 2007). Rab11 promotes the trafficking of the integrin α6β4 under hypoxic conditions, which leads to increased cell invasion of breast cancer cells (Yoon et al., 2005). Rab25 promote cell invasion into the 3D ECM by integrin α5β1 and β1 (Caswell et al., 2007). Additionally, Rab40b functions as a regulator in the transport of MMP2/9 during invadopodia formation in breast cancer (Jacob et al., 2013).

Indeed, Rab GTPases could also affect MMP inhibitors to suppress tumor invasion. For example, Rab37 controls the exocytosis of TIMP1 to inhibit inactivated MMP9 and thereby suppresses lung cancer metastasis and controls the exocytosis of TIMP2 to inhibit inactivated MMP2 in nasopharyngeal carcinoma (Li et al., 2018; Wang et al., 2018). Another protease important for the cell invasion is procathepsin L, whose secretion is regulated by Rab4a (Barbarin and Frade, 2011).

In addition, some Rab GTPases play a dual role in the regulation of cell invasion. For example, Rab7 knockdown is associated with MT1-MMP secretion and promotion of cell migration and invasion in colorectal cancer cells (Steffan et al., 2014). On the other hand, high Rab7 expression is an indicator of a higher risk of metastasis in early melanoma patient (Alonso-Curbelo et al., 2014). Another example is Rab27. Rab27a controls the secretion of MMP9 that degrades extracellular matrix proteins to promote invasion and migration (Bobrie et al., 2012). In the ER + breast cancer, Rab27b activates MMP2 secretion and stimulates breast cancer cell invasion (Hendrix et al., 2010). By contrast, Rab27a overexpressed cell-derived exosomes has been shown to suppress tumor formation *in vivo* in a mouse model, which is related to cytokines (Li et al., 2013). The opposite effect of Rab7 and Rab27 may be attributable to cancer type, stage and specific growth factor stimulation.

#### Growth Factor Driven Signaling of Migration and Invasion

The involvement of growth factor driven signaling is also essential for tumor cell invasion and migration. Rab GTPases regulate early endocytic transport of several growth factors that promote tumor cell invasion and metastasis, including epithelial growth factor (EGFR) and hepatocyte growth factor (HGF) (Porther and Barbieri, 2015). The activation of EGFR signaling and HGF signaling can activate mitogen-activated protein kinases (MAPK) signaling pathway that promote the transcription and secretion of metalloproteinases MMP2 and MMP9, actin aggregation and integrin distribution, and thus promote migration and invasion (Porther and Barbieri, 2015). In the triple-negative breast cancer, Rab5 regulate EGFR vesicular recycling to promote migration (Stallaert et al., 2018). Interestingly, Rab11a regulates EGFR circulation and promotes proliferation, but inhibits the movement in breast cancer (Yamori et al., 1981). Rab7 shRNA expressing cells were found to be more invasive, and increased invasiveness was accompanied by high activation level of HGF signaling (Steffan et al., 2014). Rab10 silencing inhibits the HGF pathway, while Rab10 overexpression indicates poor prognosis of hepatocellular carcinoma (Wang W. et al., 2017). High expression of Rab11 was closely correlated with nodal metastasis in gastric cancer tissues (Dong et al., 2016). The expression level of Rab11 was significantly increased and associated with HGF pathway. In colon cancer, increased Rab31 expression may promote tumor progression by regulating HGF secretion in the tumor stroma (Yang Y. et al., 2020). These studies suggest that vesicle trafficking modulated by Rab GTPases is involved in multiple cell invasion and migration pathways.

### Metabolism

Vesicular transport pathway and metabolic signaling pathway both involve moving substances (or signals) around the cell surface and within the cell (Chua and Tang, 2015). There is a chain of evidence to suggest that the transport and metabolic signaling pathways intersect—vesicular transport can affect the regulation of metabolic signals (Cheng et al., 2012; Wu et al., 2014; Drizyte-Miller et al., 2020). Considering Rab GTPases modulate the specificity and direction of vesicular transport, there are no doubt that they can decide the points at which vesicular transport and metabolism interact.

Rab GTPases mainly regulate the trafficking of GLUT (glucose transporter) and the formation of lipid droplet (LD) in glucose and lipid metabolism of cancer cells. GLUT1 is responsible for maintaining the basic uptake of glucose for the basic respiratory process in all cells, and responds to low levels of glucose, which increase in the cell membrane (Yang et al., 2021). Rab25 increases glycogen reserve and ATP levels in ovarian cancer cells through regulating the transport of GLUT1 to the cell surface and thus enhancing glucose uptake (Cheng et al., 2012). Although glucose metabolism is an important part of cell function, another critical aspect of cell metabolism involves lipids. A particularly salient aspect of metabolic reprogramming in tumor cells involves lipid storage and mobilization. LDs function in intracellular lipid storage, maintaining the cellular level of free lipids and energy homeostasis (Jackson, 2019). And Rab8a is reported to control lipid droplet fusion and growth in hepatocellular carcinoma (Wu et al., 2014).

The role of membrane transport in metabolic downstream events is another aspect of the regulation of metabolism by Rab GTPases, which involve mechanistic/mammalian target of rapamycin (mTOR) complex 1 (mTORC1) in downstream cascades. mTORC1 is a conserved serine/threonine kinase that regulates cell growth and metabolism in response to extracellular environmental stressors, such as aberrant nutrients, hormones, and energy (Nie et al., 2019). Rab32 knockdown increases lysosome biogenesis in hepatocellular carcinoma and cervical cancer and reduces the association of mTOR pathway proteins with lysosomes, which suggests that Rab32 regulates lysosomal mTORC1 trafficking and thereby controls metabolism (Drizyte-Miller et al., 2020). And Rab35 functions upstream of mTORC2. Oncogenic RAB35 transport PDGFRα to LAMP2-positive endomembrane without the involvement of endosomes, suggesting its oncogenic potential (Wheeler et al., 2015). Besides, other Rab GTPases, such Rab1a and Rab31 have also been reported to affect mTOR pathway in tumor, but it is unclear whether these effects are achieved by the membrane transport of Rab GTPases (Li et al., 2020a; Yang L. et al., 2020).

### Autophagy

Autophagy is a crucial cellular homeostatic process induced by nutrient deprivation that promotes the recycling of molecules from unnecessary organelles and proteins that are then employed for the synthesis of functional organelles and proteins or the energy requirement during times of need (Mele et al., 2020). The formation of autophagosomes requires vesicular trafficking from the subcellular compartment to the site of autophagosome formation (Onorati et al., 2018). Therefore, a series of Rab GTPases play crucial roles in this process (Szatmari and Sass, 2014). For example, the Optineurin-Rab1a complexes modulate autophagosome formation through regulating the translocation of LC3-EGFP to autophagosomes (Song et al., 2018). Both Rab2 and Rab7 are important to autophagosome maturation and autolysosome fusion, which indicates that Rab2 and Rab7 are key regulators in the delivery and degradation of autophagic cargos (Lorincz et al., 2017). In addition, RAB2 regulates the activity of ULK1 kinase, thus promotes autophagosome formation (Ding et al., 2019).

Indeed, the selective autophagic degradation of LDs is called lipophagy (Singh et al., 2009; Galluzzi et al., 2014). Thus, lipophagy represents the intersection of fat metabolism and autophagy. In this process, LDs bind autophagosomes and are moved into lysosomes, and Rab GTPases function in membrane trafficking. Rab10 could induce the recruitment of autophagosomes at the LD surface in hepatocellular carcinoma (Li et al., 2016, 2020b). In addition, Rab7 regulates the transport of multivesicular bodies and lysosomes to LDs in hepatocellular carcinoma (Schroeder et al., 2015).

Autophagy plays a dual role in cancer, depending on the cellular context and the extracellular environment of the tumor (Onorati et al., 2018). Notably, specific Rab GTPases also have dual functions in different types or subtypes of cancer. For example, in ovarian cancer cells, Rab25 knockdown increases autophagy levels and induces apoptosis. Rab25 promotes cancer in ovarian cancer by inhibiting autophagy (Liu et al., 2012). However, increased expression of Rab25 inhibited the FAK/Akt pathway, promoted autophagy, and inhibited the malignancy of renal carcinoma cells (Tringali et al., 2012). The above results suggest that autophagy may be involved in the mechanism of the dual roles of Rab GTPases in different tumors.

### Exosome Biogenesis and Secretion

In our previous studies, we discussed the characteristics and mechanism of exosome secretion in cancer (Fan et al., 2018; Yang et al., 2019). Exosomes, nanoscale extracellular vesicles 40–100 nm in size, have attracted increasing attention because of their involvement in intracellular communication. Exosomes are derived from multivesicular bodies (MVBs). MVBs are late endosomal structures containing luminal vesicles. After the fusion of MVBs with the plasma membrane, exosomes are released.

The Rab GTPases have been observed to significantly promote exosome biogenesis and secretion. Reduced activity of RAB11 in K562 cells is associated with reduced exosome release (Savina et al., 2002). Rab11 is also involved in the interaction between MVB and autophagosomes in K562 cells. Rab11 co-locates with LC3 during autophagy, which is associated with reduced exosome release. Further studies showed that Rab11 is involved in the docking of Ca^2^^+^ dependent MVB to the plasma membrane (Savina et al., 2005). Rab11-mediated secretion of exosomal TMPRSS2 promoted cell migration in breast cancer (Chi et al., 2020).

Although Rab11 is critical for exosome release in K562 cells and breast cancer cells, Rab11 does not affect exosome secretion in HeLa cells. In HeLa cells, the silencing of Rab27a and Rab27b reduces exosome secretion (Ostrowski et al., 2010). Rab27a regulates the size of MVB, while Rab27b controls their cell localization.

Invadopodia are an important site of Rab27a-regulated exosome secretion (Hoshino et al., 2013). So the secretion of Rab27a and exosomes is intrinsically related to the invasion of cancer cells. Rab27a knockdown in invasive cancer cell lines decreased exosome secretion, and Rab27a-mediated exosomes increased cell migration, chemotaxis, and invasion (Hendrix and De Wever, 2013; Chen et al., 2018; Guo et al., 2019). Furthermore, the transfer of mRNA into recipient cells was demonstrated to occur via exosome secretion by Rab27 regulation. Rab27-mediated secretion of exosomal miR-23b was shown to be connected with invasion and metastasis in bladder carcinoma (Ostenfeld et al., 2014). Rab27b-regulated exosomal miR-34c-5p was demonstrated to be related to the senescence of leukemia stem cells (Peng et al., 2018). Although Rab35 are also reported, the function of Rab35-regulated exosomes in cancer remains unclear (Yang et al., 2019).

In addition to regulating tumor cells, Rab27a/b also participates in exosome exchange between different cells in the tumor microenvironment. Rab27a-induced exosomes derived from hypoxic colorectal cancer cells affected the growth and invasion of endothelial cells (Huang and Feng, 2017). Both Rab27a/b gene deletions in head and neck squamous cell carcinoma cells reduce exosomal-mediated innervation induction (Madeo et al., 2018). Similarly, exosomes derived from Rab27a-overexpressing cancer cells elicited the efficient induction of antitumor immunity (Li et al., 2013). Rab27a/b also regulates macrophage exosome secretion (Binenbaum et al., 2018). Co-secretion of Rab27a-dependent exosomes contributes to the mobilization of tumor-promoting neutrophils and supports the growth of mouse breast tumors and their metastasis to the lung (Bobrie et al., 2012). It is worth noting that the role of Rab11 and Rab27a/b in exosome secretion is mainly based on *in vitro* experiments, and it is unclear whether Rab11 and Rab27a/b have similar functions *in vivo*.

### Drug Resistance

Rab GTPases function in tumor drug resistance mainly from the following four aspects: (i) Rab7a affects drug efflux by the extracellular vesicle (Guerra et al., 2019). The exposure of cancer cells to hypoxia increased their exosome-medicated cisplatin efflux by upregulation of Rab27a (Dorayappan et al., 2018). These findings indicated the close relationship between Rab-regulated exosome secretion and tumor drug resistance. (ii) Rab8 can cooperate with transmembrane transport protein to promote drug resistance. Human epidermoid carcinoma resistant to cisplatin exhibits high Rab8 level. Rab8 colocalize with the TMEM205 and increase the secretion of cisplatin (Shen and Gottesman, 2012). (iii) Some Rab GTPases promote drug resistance by transporting membrane surface specific receptors. TNFRSF10B expression levels and cell surface levels are increased after treatment with certain chemotherapeutic agents, such as pemetrexet, which plays a key role in induction of apoptosis. Rab8 suppresses pemetrexet effect by regulating the TNFRSF10B transport to the cytoplasm (Wang et al., 2020). Besides, Rab13 affect the sensitization of gastric cancer to 5-fluorouracil treatment by regulating the EGFR transport to the cell membrane (Chen et al., 2019). (iv) Rab5a and Rab21 function through recycling multidrug resistance (MDR) relative protein. ABCG2 (ATP-binding cassette transporter of subfamily G) is a half-transporter involved in drug efflux and the development of MDR in cancer cells (Pasello et al., 2020). Ectopically expressed ABCG2 was shown to be located in cell surface and function in drug efflux (Kapoor et al., 2018). Active Rab5a-Q79L in breast cancer cells reduced the expression of ABCG2 in the plasma membrane, decreasing ABCG2-mediated drug efflux. Moreover, a reduction in the Rab21 expression level promoted the surface localization of ABCG2. Rab5a and Rab21 function in ABCG2 surface localization and turnover and may be targets to overcome MDR (Yousaf and Ali, 2020).

## Clinical Implications of Rab GTPases in Cancer

Many clinicopathological studies note that Rab GTPases function in several cancers (Goldenring, 2013). Here, we discuss the clinical significance of the Rab GTPases mentioned above (Table 2). Rab1a expression is associated with tumor size, differentiation, lymph node metastasis, TNM stage and poor prognosis in patients with tongue squamous carcinoma, hepatocellular carcinomas and breast, prostate, lung, gastric, colorectal cancer (Cheng et al., 2019; Shao et al., 2019a,b). Thus, low Rab1b expression correlates with poor prognosis of breast cancer patients (Jiang et al., 2015). Rab2 is associated with poor prognosis of pancreatic and breast cancer patients (Luo et al., 2015; Jin et al., 2018). Rab5a is essential for the formation of vesicles that are specifically degraded by the extracellular matrix. Therefore, Rab5a was necessary to promote the local invasion and long-distance transmission of tumor cell lines, and this metastatic ability was associated with increased cellular motility. Therefore, high Rab5 expression correlates with poor prognosis in several cancers, including lung, liver, breast, ovarian cancer and glioma (Frittoli et al., 2014; Jian et al., 2020). Rab7 is an early-induced melanoma driver (Alonso-Curbelo et al., 2014). In addition, lung cancer and gastric cancer patients with high Rab7 expression show decreased survival rates (Liu et al., 2020; Xiao and Schmid, 2020). High Rab10 expression levels are associated with poor prognosis in HCC patients (Wang W. et al., 2017). In addition, colorectal carcinoma patients with high expression of both E-cadherin and Rab11 show a poor prognosis (Chung et al., 2016). Furthermore, Rab11a is associated with advanced TNM stage, positive nodal status and poor prognosis (Dong et al., 2017). Rab13 can induce chemotherapeutic resistance and indicates poor overall survival and progression-free survival (Chen et al., 2019). Rab21 is also highly expressed and associated with poor prognosis in pancreatic cancer (Anand et al., 2020). Low Rab37 protein expression levels in lung cancer indicate poor prognosis in patients with lung cancer at different stages and lymph node metastasis (Tsai et al., 2014). Additionally, Rab40b correlated with the prognosis, invasion classification, lymph node metastasis, and pathological stage (Li et al., 2015).

**TABLE 2:** Clinical implications of Rab GTPases in cancer.

**Rab GTPase**	**Cancer types**	**Expression**	**Clinical implications**	**References**
Rab1a	Tongue Breast Prostate Liver Lung Stomach Colorectum	Increased	Poor prognosis	Cheng et al., 2019; Shao et al., 2019a,b
Rab1b	Breast	Decreased	Good prognosis	Jiang et al., 2015
Rab2	Pancreas Breast	Increased	Poor prognosis	Luo et al., 2015; Jin et al., 2018
Rab5a	Lung Liver Breast Ovarian Glioma	Increased	Poor prognosis	Frittoli et al., 2014; Jian et al., 2020
Rab7	Melanoma Lung Stomach	Increased	Poor prognosis	Alonso-Curbelo et al., 2014; Liu et al., 2020; Xiao and Schmid, 2020
Rab10	Liver	Increased	Poor prognosis	Wang W. et al., 2017
Rab11 Rab11a	Colorectum Lung	Increased	Poor prognosis	Chung et al., 2016; Dong et al., 2017
Rab13	Stomach	Increased	Poor prognosis	Chen et al., 2019
Rab21	Pancreas	Increased	Poor prognosis	Anand et al., 2020
Rab25	Breast Ovarian Kidney Lung Stomach Liver Bladder Glioma Prostate	Increased	Poor prognosis	Wang S. et al., 2017
	Colorectum Esophagus Head and neck	Decreased	Poor prognosis Good prognosis	Nam et al., 2010; Wang S. et al., 2017
Rab27a	Liver	Increased	Poor prognosis	Guerra et al., 2019
	Colorectum Prostate	Decreased	Good prognosis	Dong et al., 2015; Worst et al., 2017
Rab27b	Bladder Breast Liver	Increased	Poor prognosis	Hendrix et al., 2010; Zhang et al., 2012; Ostenfeld et al., 2014; Binenbaum et al., 2018; Madeo et al., 2018; Guerra et al., 2019
	Prostate	Decreased	Good prognosis	Worst et al., 2017
Rab37	Lung	Increased	Poor prognosis	Tsai et al., 2014
Rab40b	Stomach	Increased	Poor prognosis	Li et al., 2015

Sometimes, the role of Rab GTPases is different according to tumor type. The role of Rab GTPases is sometimes influenced by tumor type. Elevated expression of Rab27b is correlated with reduced survival times, lymph node metastasis and pathological grade (Hendrix et al., 2010; Zhang et al., 2012; Ostenfeld et al., 2014). Rab27a and Rab27b was associated with poor patient prognosis and advanced TNM stage (Dong et al., 2012). Although Rab27 seems oncogenic, it also functions as a cancer inhibitor. Rab27a is downregulated and correlated with poor patient prognosis, advanced TNM stage, distant metastasis, and local recurrence (Dong et al., 2015). Low expression of Rab27a and Rab27b is also correlated with poor prostate cancer patient prognosis (Worst et al., 2017). Another example is Rab25, which functions as a prognostic indicator for breast, ovarian, kidney and other cancers. However, low expression of Rab25 is correlated with a poor prognosis in colon cancer, suggesting its role in tumor inhibition (Nam et al., 2010). The significant conflict between the functions of Rab25 in promoting or inhibiting cancer progression in different cancer types might be due to the involvement CLIC3, which is needed for RAB25-regulated integrin transport (Wang S. et al., 2017).

## Targeting Rab GTPases as a Cancer Therapeutic Strategy

Rab GTPases are widely expressed and function as prognostic markers in various human cancers. Therefore, targeting Rab GTPases is undoubtedly a highly attractive strategy for drug discovery, and some efforts to target Rab GTPases have already been made. For example, ML282 can inhibit Rab7 with high efficacy (Hong et al., 2010). In addition, the use of competitive inhibitors that bind nucleotides is a feasible method to target Rab GTPases. Some small-molecule pan-GTPase inhibitors, such as CID1067700, were discovered by high-throughput screening experiments. CID1067700 could inhibit Rab7 in biochemical, cellular protein and downstream protein interaction assays, and cellular functional assays (Hong et al., 2015). Although CID1067700 and ML282 also inhibit other GTPases, pan-GTPase inhibitors could serve as templates from which to develop specific Rab GTPase inhibitors with drug potential.

Gene therapeutics targeting Rab GTPase have recently been utilized. Once the Rab GTPases that are crucial for progression were established, their expression could be affected by small interfering RNA as well as non-coding RNA (ncRNA), such as long non-coding RNA (lncRNA) and microRNA (miRNA). For example, lncRNA HOTAIR could regulate Rab35 expression and localization, and thereby affect the exosome secretion in hepatocellular carcinoma (Yang et al., 2019). LINC00152 function as a sponge for miR-107 that targets Rab10 directly to promote tumor progression (Zhou and Huang, 2019). LncRNA SNHG3 also functions as a sponge in the regulation of miRNA-151a-3p/Rab22a to modulate migration of osteosarcoma (Zheng et al., 2019). Although these studies have suggested that the suppression of oncogenic ncRNAs and overexpression of tumor-inhibited ncRNAs might serve as attractive methods for therapeutic schedule, improving ncRNAs delivery systems, ncRNAs stability and off-target effects remains a challenge. If we want to successfully achieve the translation of gene therapeutics from the bench to the bedside, these questions must be addressed.

## Conclusion

Vesicle trafficking functions in invasion, migration, metabolism, autophagy, exosome secretion and drug resistance. Rab GTPases are central coordinators of membrane trafficking and act as critical checkpoints for vesicular transport. Furthermore, small alterations in Rab GTPases may cause significant changes in net trafficking. Because membrane recycling is tremendously dynamic, one cycle of protein recycling might take only a few minutes or even less time. Therefore, the abnormal expression of Rab GTPases can markedly affect vesicle trafficking. A small change in the expression or active state of Rab GTPases might cause large changes in cell processes over days and thereby might promote invasion, migration, metabolism, autophagy, exosome secretion and drug resistance. Overall, Rab GTPases are essential to cancer progression, and selective targeting of particular Rab GTPases might be an attractive therapeutic schedule. Strategies to change membrane trafficking through targeting Rab GTPases might help to provide therapeutic approaches to reverse tumor invasion, migration, metabolism, autophagy, exosome secretion and drug resistance.

## Author Contributions

HJ and YT designed the review article. LY, XP, BL, QF, SW, SY, XL, BW, MH, ST, and JL performed literature collection. HL conceived the study. All authors read and approved the final manuscript.

## Conflict of Interest

The authors declare that the research was conducted in the absence of any commercial or financial relationships that could be construed as a potential conflict of interest.
